# Risk Factors and Predictors of Prolonged Mechanical Ventilation Following Cardiac Surgery: A Narrative Review

**DOI:** 10.7759/cureus.68011

**Published:** 2024-08-28

**Authors:** Kartik Bhagat

**Affiliations:** 1 Internal Medicine, Tbilisi State Medical University, Tbilisi, GEO

**Keywords:** ventilator weaning, prediction models, pulmonary critical care, spontaneous breathing trial, adult cardiac surgery, prolonged mechanical ventilation

## Abstract

The subset of patients requiring prolonged mechanical ventilation is significantly high worldwide, making it an important topic of continuous and ongoing research. Over the years, various articles have shown that there may be predictors of prolonged ventilation that could be applied in healthcare to make it more patient-centered. The available literature suggests that authors have different definitions of “prolonged” ventilation. However, most critical care units embrace caution if a patient needs mechanical ventilation for more than 48 to 72 hours. The major benefits of mechanical ventilation are an overall decrease in the work of breathing and the facilitation of relatively easier pumping from an ailing heart. An elevated risk of prolonged ventilation after cardiac surgery exists in patients with higher classes of heart failure (as classified by the New York Heart Association (NYHA) or Canadian Cardiovascular Society (CCS)), a pre-existing congenital or acquired cardiac abnormality, and patients with renal failure, to name a few. The impact on quality of life has also been widely studied; as mortality rates increase with factors like age and days dependent on ventilation. Patients undergoing prolonged ventilation constitute an administrative challenge for critical care units, highlighting how multiple patients in this bracket can overwhelm the healthcare system. The use of prediction models in this context can aid healthcare delivery tremendously. Using different predictors, we can craft tailor-made treatment options and achieve the goal of more ventilator-free days per patient.

## Introduction and background

In one of the earliest (and pioneering) articles dating back to 1981, Dr. Alan Gilston conducted a retrospective study comprising 25 patients. These patients required prolonged mechanical ventilation following cardiac surgery for both congenital and acquired heart diseases [1]. He concluded that pre-existing cardiac disease coupled with post-surgical heart failure facilitated the onset of respiratory distress in said patients, but also mentioned that its emergence is not always clear. The advent of technology in the early 2000s and its synergy with healthcare have blessed every avenue of medicine, giving doctors the necessary means to monitor their patients by the second. Since then, numerous critical care physicians and cardiac surgeons have published their findings about the topic, aiding doctors globally through the means of open-access journals. This literature review is a comprehensive evaluation of the studies conducted over the years highlighting the risk factors that may necessitate prolonged mechanical ventilation after open cardiac surgery. I hope this paper can contribute to the continuously evolving network of literature on the topic, which can further aid young students and doctors globally.

It is well-known that patients on mechanical ventilation have significantly higher rates of morbidity and mortality. Over the years, a pool of literature has been published on the topic, detailing cases, guidelines, and prediction models from various hospitals globally. In ICUs, the type of surgical procedure and local protocols (according to which a patient is intubated) also differ. Therefore, prolonged ventilation is not uniquely defined. Gilston defined the term prolonged ventilation by using set criteria. These criteria included a minimum requirement of ventilator therapy for three days and clinical signs of respiratory distress if mechanical ventilation is interrupted [1]. Another paper by Pappalardo et al. defined it as “a patient needing mechanical ventilation for ≥7 days” [2]. A relatively simplistic approach was put forward via a consensus by Boles et al.: easy weaning, difficult weaning, and prolonged weaning [3]. If a single, successful attempt at separation from mechanical ventilation allows for spontaneous breathing, it is termed easy weaning. Difficult weaning is defined as a failed first separation attempt, requiring additional spontaneous breathing trials (SBTs) essential to extubate. When patients need more than three SBTs or seven or more days of ventilation after the first SBT, it is termed prolonged weaning. An international consensus defined prolonged ventilation as at least six hours of ventilation per day for 21 consecutive days [4]. However, most critical care units embrace caution if a patient needs mechanical ventilation for more than 48 to 72 hours.

**Figure 1:**
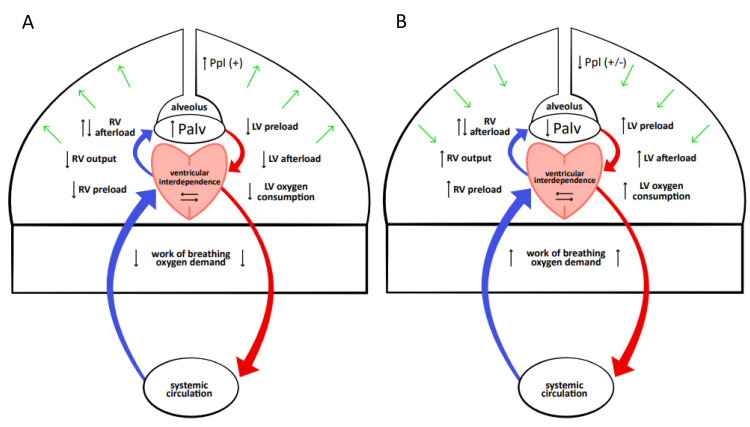
Effects of Mechanical Ventilation and Weaning on Cardiorespiratory Physiology The myriad of effects caused by (A) positive pressure ventilation and (B) weaning/SBT. Ppl: intrapleural pressure; Palv: alveolar pressure; RV: right ventricle; LV: left ventricle; SBT: spontaneous breathing trial

To form a comprehensive review of the topic, establishing a relationship between mechanical ventilation and cardiac output is important. In his paper on prolonged mechanical ventilation, Dr. Alan Gilston concluded, “Derangement of lung anatomy and mechanics is of much greater practical importance than deterioration of pulmonary gas exchange in the context of ventilator therapy and survival” [1]. During prolonged ventilation, the deterioration of the structure and anatomy is more significant than its impact on alveolar physiology and gaseous diffusion processes. This can be explained by visualizing the effect of positive pressure ventilation on the thoracic cavity (Figure 1), and its impact on cardiorespiratory effort.

• When a patient is intubated (Figure 1A), positive pressure ventilation increases the intrapleural and alveolar pressures. This continuous pressure causes severe alveolar distention (theoretically here the pulmonary capillaries are slightly compressed), but thorough oxygenation of the vascular bed reduces hypoxic vasoconstriction. A tricky yet fine balance between the two allows for a net effect of decreased pulmonary vascular resistance, decreased right ventricular workload, and lower output. Post pulmonary circulation, there is a concurrent decrease in left ventricular preload and afterload; thereby decreasing left ventricular myocardial oxygen consumption. Additionally, positive pressure reduces the work of breathing, decreasing oxygen demand. Furthermore, the sedation typically associated with intubation tends to lower the sympathetic tone and consequently the systemic vascular resistance and heart rate. Therefore, the above-mentioned processes contribute a multifactorial aid to the heart and lungs.

• On extubation (or during SBTs) (Figure 1B), the weaning of positive intrathoracic pressure increases cardiac workload. Additionally, a significant increase in the global oxygen demand is seen due to an increased work of breathing, decreased sedatives, stress, and anxiety related to extubation. Therefore, the weaning process may lead to myocardial ischemia, acute ventricular dilation, functional mitral regurgitation, and finally heart failure [5,6], eventually manifesting as weaning-induced pulmonary edema (WiPO).

## Review

Methods

A detailed search was carried out on PubMed and the National Institute of Health database. References of relevant papers were also explored and a manual search was carried out to expand the spectrum of the literature reviewed. Inclusion criteria are as follows: (1) Articles on cardiac surgery and mechanical ventilation; (2) Literature from 1980 to 2023; (3) Keywords - “Prolonged Mechanical Ventilation”, “Cardiac Surgery”, “Positive Pressure Ventilation”, “Hemodynamics of Weaning”, “ Spontaneous Breathing Trials”. Exclusion criteria are as follows: (1) Literature about concurrent organic/primary lung and heart pathology; (2) Articles including a pediatric population.

Results

Data collected over time suggests that most patients recover spontaneously from cardiac surgery and do not require any ventilator therapy during the postoperative period. However, the minority of patients who fail to wean from ventilation can measure from 2.6% to 22.7% [7]. Prolonged ventilation for ≥72 hours has a strong positive correlation with a negative outcome with mortality rates as high as 25-50% [8,9]. A prospective observational study from Milan concluded that among patients requiring mechanical ventilation ≥21 days, long-term survival was severely affected. The one-year survival rate was found to be 70.9% and the five-year survival rate was 64.5%. Insignificant or no limitation in daily living was mentioned by 69% of the survivors [2]. Prolonged ventilation also piles up healthcare expenses, up to 18 times in comparison with patients undergoing early extubation [7,10].

Surgeons and critical care doctors have stated several risk factors in numerous publications, but only a few have been reported consistently. The following sets of patients are at a much higher risk for prolonged ventilation after cardiac surgery - (1) those with higher classes of heart failure according to the New York Heart Association and the Canadian Cardiac Society; (2) patients in a state of shock or delivering low cardiac output; (3) those with renal failure; (4) those needing inotropic support; (5) varying risk depending on the type and timing of the surgery [6,11]. Special attention must be paid to these patients but the risk of requiring mechanical ventilation or its prolonging is not limited to these factors. Other literature suggests that the need for blood transfusion, body mass index, the duration of cardiopulmonary bypass, presence of arrhythmias (non-specific to the origin of the arrhythmia), organic lung disorders, and chronic obstructive pulmonary disease must also be taken into account while predicting prolonged ventilation [9,10,12].

Prediction models (see “Discussions”) also allow surgeons and critical care doctors to better anticipate the onset of postoperative complications as well as successful weaning. At a cohort in an intensive care center in Poland, Knapik et al. found aortic aneurysm surgery, combined procedures, valve procedures, preoperative renal dysfunction, and preoperative stroke to be important predictors. These predictors may be useful if incorporated into prediction models [13].

Since weaning failure is not subject to just cardiac causes, a thorough evaluation of the patient’s condition is required. A holistic approach must be pursued and the pathophysiology must be comprehensively studied. Every member of the critical care team must understand that the condition of the patient isn’t necessarily the result of a singular event, but rather a multifactorial process. In a multivariable logistic regression analysis conducted by Trouillet et al., factors associated with successful weaning from mechanical ventilation on postoperative day 10 were studied. The highest odds for successful weaning were seen in patients who had a urinary output of >500 mL/24 hours [7].

As discussed further, other significant predictors of unsuccessful weaning can be (but are not limited to) pulmonary artery occlusion pressure (PAOP), biomarkers indicating cardiac failure, ultrasound, and nutrition.

Discussions

Gilston had established early on that mechanical ventilation helped alleviate the increase in respiratory work and pulmonary hypertension. Additionally, he discussed that in terms of clinical signs and mortality respiratory distress after cardiac surgery resembles classic respiratory distress syndrome (RDS). Despite this, he was unable to explain the absence of the typical “white-out” appearance of RDS in more than 80% of the patients in his study. Another aggravating factor was the onset of sepsis; seen in 50% of the fatal cases in his study [1].

According to Knapik et al., global healthcare has evolved more in the management of acutely sick and elderly patients with heart failure, demanding more urgent procedures. The percentage of emergency cases has risen by almost 13% [13]. Thus, urgent surgery is now the rule and not the exception. Cardiopulmonary bypass surgery was once performed on difficult and critical cardiac patients whereas off-pump coronary artery bypass (OPCAB) was the norm to treat more direct cardiac surgery cases. But now, the tables have turned and the OPCAB procedure is reserved for more elderly patients with co-morbidities [14].

With a boom in the med-tech industry, a topic of great interest is the use of automatic weaning modes. While predicting the success of any weaning procedure, it is important to factor in the body’s ability to adapt to the lack of artificial ventilation after weaning. Automated closed-loop systems offer a solution to this. An input (such as end-tidal CO_2_) into a protocol activates the system through which an output (such as an adjustment in positive pressure) is generated. The system continuously monitors a change in various inputs and helps adapt ventilation [15].

It is also widely understood that a patient ventilated for ≥48 hours will prove to be a managerial challenge for any critical care department [13]. If we extrapolate this to a national level, multiple patients over time may fall into this subset of the population and overwhelm the healthcare system. A smaller proportion of patients may be on ventilator support for months or years. In situations like these, it may be crucial to evaluate the use of active euthanasia. This could alleviate the ongoing burden on hospitals. But primarily, it may provide relief to the patient's family and friends from the financial and emotional strain of prolonged suffering.

Further Predictors

Brain natriuretic peptide (BNP) - a cardiac marker as a tool: If we revisit Gilston’s paper, he makes an interesting observation that was less discussed during his time. He observed and concluded that the patients in his study did not die from hypoxemia, but from heart failure [1]. Since mechanical ventilation alleviates the load on the pulmonary and cardiovascular systems, cardiac biomarkers have proven to be useful in the evaluation and prediction of a successful weaning procedure. Recent studies have shown a positive correlation between elevated levels of BNP and weaning failure [16,17]. BNP-driven fluid management strategies using diuretics have also been associated with a decrease in the duration of ventilator usage [18].

Ultrasonographic evaluation: Ultrasonography has also proven to be a beneficial tool in predicting patient outcomes during weaning procedures. The overall extent of diastolic dysfunction, visualization of the mitral valve, and in particular the maximum speed of the mitral E' wave have been of immense significance in predicting successful weaning, all of which are possible only through a cardiac ultrasound [19,20]. Lung and diaphragm ultrasound also serve as important tools in predicting outcomes and they were of great clinical significance during the SARS-CoV-2 pandemic [21,22].

Nutritional status: In hospitals, nutritional status is usually judged according to parameters such as BMI and albumin levels. A positive correlation has been noted between preoperative malnutrition, prolonged mechanical ventilation, and multiple postoperative complications [23]. Several retrospective studies have also demonstrated that a higher protein and caloric intake is linked with successful weaning [24,25]. Studies have shown varying effects of supplementation of the amino acid glutamine. A meta-analysis conducted by Chen et al. concluded that glutamine supplementation reduces nosocomial infections and provides a great deal of benefit to surgical patients. However, glutamine supplementation does not directly contribute to decreased mortality [26]. A limitation of the above-mentioned studies is that they were conducted across several departments and not just in cardiac units. Nonetheless, appropriate nutrition must be deliberated in patients undergoing difficulties during weaning.

PAOP: As ventilator therapy and weaning have significant hemodynamic implications, PAOP may increase within minutes following the withdrawal of ventilator support (also during SBTs). It has been previously described that a PAOP of >18 mmHg is categorized as pulmonary edema secondary to cardiac failure [11]. According to Nassar et al., a PAOP >18 mmHg correlates with weaning failure but a PAOP <15 mmHg does not correlate with weaning failure [27]. Regardless of the above-mentioned findings, multiple predictors must be involved to accurately predict outcomes. A single predictor may have limitations and may be subject to confounding factors that warrant further studies.

Tracheostomy - A Blessing for Ventilated Patients?

As prolonged ventilation brings numerous complications in healthcare delivery and survival rates, a tracheostomy may help in reducing these complications. It prevents trauma to the vocal cords from the endotracheal tube and reduces airway resistance. If an early tracheostomy is done, it may also decrease neurological complications associated with prolonged sedation. But above all, a tracheostomy reduces the work of breathing which in turn facilitates quicker weaning. However, strong data regarding the impact of tracheostomies on clinical outcomes is scarce [28].

In a retrospective observational study, Okada et al. concluded that a tracheostomy done within seven days of critical care admission led to fewer complications, better outcomes, and lower mortality and morbidity rates [29]. Another retrospective observational study by Affronti et al. found that a tracheostomy done <14 days after cardiac surgery led to shorter ventilation times and decreased ICU length of stay [30].

Prediction Models and Patient Care

The majority of the discussion in this review has been dedicated to identifying predictors of prolonged ventilation and its correlation with favorable clinical outcomes. We must describe how these predictors can be used by physicians to better deliver quality health care. A collection of excellent predictors can shape useful prediction models which in turn improve patient-centered care. This shall also allow doctors to curate a more personalized treatment plan across all branches of medicine.

However, a revolution in patient care has also been paralleled with an evolution in disease processes. Note that prediction models may vary across different departments and must be updated when major changes in patient management are necessary. This can be done by analyzing patient data via clinical audits and retrospective studies, revealing the incidence of particular complications over the preceding few years. It will be of great interest to note how emerging, evidence-based predictors of postoperative complications contribute to the development of prediction models aimed at enhancing the quality of care.

## Conclusions

Abundant literature on mechanical ventilation serves as proof that physicians have worked extensively to prevent prolonged ventilation and its complications. This review focuses on prolonged ventilation in cardiac surgery patients. A thorough understanding of the effects of mechanical ventilation on cardiovascular physiology and hemodynamics is extremely important. Additionally, a few risk factors and predictors that determine clinical outcomes are detailed. Notable risk factors are the severity of heart failure and the presence of renal failure before surgery, among others. A matter of key concern to critical care departments is the organizational challenge that accompanies any patient dependent on ventilation for >48 hours. Hence, the hospital-wide use of various prediction models must be encouraged to provide better overall care. New predictors of prolonged mechanical ventilation will be of strategic interest to ICUs and their application in clinical settings might prove to be beneficial.
